# Clinical Utility of Serum Holotranscobalamin Measurements in Patients with First-Ever Ischemic Stroke

**DOI:** 10.1155/2021/9914298

**Published:** 2021-09-10

**Authors:** Oh Joo Kweon, Yong Kwan Lim, Mi-Kyung Lee, Hye Ryoun Kim

**Affiliations:** Department of Laboratory Medicine, Chung-Ang University College of Medicine, Seoul, Republic of Korea

## Abstract

**Background:**

Whether holotranscobalamin (holoTC) indicates B12 deficiency more sensitively than total vitamin B12 (B12) is unclear. This study is aimed at determining the impact of serum holoTC level as a risk factor for ischemic stroke and investigating its association with disease severity and short-term outcomes.

**Methods:**

Serum holoTC, total B12, and homocysteine levels were compared between 130 stroke patients and 138 healthy controls. Biomarker level correlations with disease severity and stroke functional outcomes were investigated.

**Results:**

holoTC levels were lower and homocysteine levels were higher in stroke patients than in healthy controls (*P* < 0.05). The holoTC/total B12 ratio and homocysteine level significantly predicted ischemic stroke in the multivariable regression analysis (*P* < 0.05). Along with hyperhomocysteinemia, patients more often had holoTC than total B12 deficiency (6.2% vs. 3.1%). holoTC levels negatively correlated with homocysteine levels (partial *R* -0.165, *P* < 0.05) in stroke patients in multiple linear regression analyses, but not total B12 levels. The holoTC level and holoTC/total B12 ratio, but not homocysteine and total B12 levels, negatively correlated with the National Institute of Health Stroke Scale (partial *R*, -0.405 and -0.207, respectively, *P* < 0.01).

**Conclusions:**

Measurements of serum holoTC levels combined with total B12 and homocysteine levels may provide valuable information for predicting ischemic stroke and its severity and short-term outcomes of ischemic stroke patients.

## 1. Introduction

Stroke is the second most common cause of death worldwide and is the leading cause of acquired disability in adults in most regions [1]. Ischemic stroke has potentially treatable contributory factors such as hypertension, diabetes mellitus, cardiac diseases, smoking, alcohol intake, obesity, and hyperlipidemia [2, 3]. In addition to these, inadequate vitamin B12 or cobalamin status has emerged as a new treatable factor contributing to stroke risk, implying that early detection of vitamin B12 deficiency is crucial [4, 5].

Vitamin B12 is essential for cellular metabolism, particularly for DNA synthesis and both fatty acid and amino acid metabolism. Importantly, vitamin B12 is a cofactor in homocysteine metabolism, and its deficiency may be an important cause of hyperhomocysteinemia [6, 7], which is closely correlated to the risk of ischemic stroke as a result of the increased development of carotid plaques [2]. Vitamin B12 supplementation may contribute to stroke prevention [8], thus lowering homocysteine levels.

Currently, vitamin B12 status is mainly determined by measuring total levels of vitamin B12 in the serum (total B12), but the diagnostic accuracy of these tests is not satisfactory for detecting vitamin B12 deficiency [4, 9]. This could be because total B12 assays detect both active and inert forms of vitamin B12. Only 20–25% of serum total B12 is in the active form, i.e., holotranscobalamin (holoTC). holoTC is vitamin B12 that is bound to the serum transport protein transcobalamin for delivery to cells for metabolism. Total B12 measurement can, therefore, be insensitive in detecting functional vitamin B12 deficiency [10, 11]. Another potentially accurate way to diagnose the functional adequacy of vitamin B12 is to measure the level of holoTC [9, 12]. However, it remains controversial whether holoTC values provide an earlier or more sensitive indication of vitamin B12 deficiency than total B12 values as the results of different studies have been inconsistent [11].

The clinical usefulness of total B12 measurements in ischemic stroke patients has been assessed previously [2]; however, few studies have investigated the clinical utility of holoTC measurements for this patient population. This study is aimed at determining the impact of serum holoTC level as a risk factor for ischemic stroke and investigating its association with disease severity and short-term outcomes, focusing on the advantages, if any, of holoTC measurements over total B12 measurements.

## 2. Materials and Methods

### 2.1. Ethics Statements

This prospective case-control study was approved by the Chung-Ang University Hospital Institutional Review Board (IRB), and the need for informed consent was waived according to the IRB policy (IRB approval number 1712-022-16129).

### 2.2. Study Subjects

**Figure 1:**
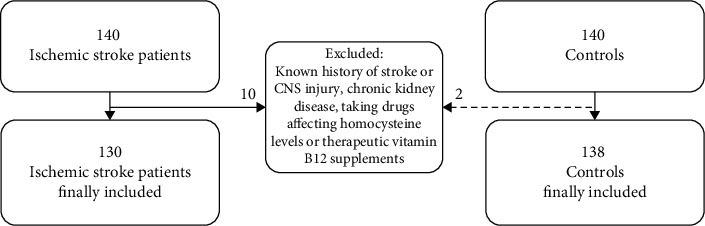
Flowchart of the inclusion and exclusion of the study subjects. In total, 130 ischemic stroke patients and 138 normal controls were included in this study. Patients who previously had an injury to the central nervous system or who had experienced stroke events were excluded. Patients taking therapeutic vitamin B12 supplements for other diseases such as gastric cancer and with factors affecting serum homocysteine levels (chronic kidney disease and certain drugs) were also excluded.

The study included first-ever ischemic stroke patients who experienced acute neurologic symptoms with symptom onset within 24 h and visited the emergency room of Chung-Ang University Hospital, Seoul, Republic of Korea, from August 2018 to February 2019. A total of 130 stroke patients (81 males) were included (Figure 1). The diagnosis of ischemic stroke was confirmed based on findings from diffuse brain magnetic resonance imaging (MRI) according to the American Heart Association/American Stroke Association definition [13]. In addition to the stroke patients, 138 (88 males) healthy individuals who visited the hospital for health check-ups were enrolled in the study as healthy controls. The healthy controls were screened for the absence of stroke using brain MRI.

Patients diagnosed with hemorrhagic stroke, who previously had an injury to the central nervous system or who experienced cerebro- or cardiovascular ischemic events, were excluded from the study. To limit factors affecting the circulating homocysteine level, patients with chronic kidney disease and receiving certain drugs such as fibrates, metformin, and methotrexate were excluded. Patients who took therapeutic vitamin B12 supplements for other diseases such as gastric cancer were also excluded. Patients who had been taking nutritional dietary supplements such as vitamin B12 before the onset of stroke were not excluded and their B12 supplementation was discontinued during their hospitalization period.

### 2.3. Laboratory Data

Serum levels of holoTC, total B12, homocysteine, hemoglobin A1c (HbA1c), fasting glucose, and lipids (total cholesterol, LDL cholesterol, and triglycerides) were measured using the blood samples obtained from ischemic patients at the time of diagnosis and obtained from healthy controls during their visit for health check-ups. holoTC levels were assessed using an Abbott Architect Active-B12 (holotranscobalamin) assay (Abbott Laboratories, Abbott Park, IL, USA) with an ARCHITECT i2000SR (Abbott Laboratories), which is an automated chemiluminescent microparticle immunoassay analyzer. Total B12 levels were measured using radioimmunoassay (MP Biomedicals, Orangeburg, NY, USA). The holoTC/total B12 ratio was calculated after unifying the units and was expressed as a percentage. Homocysteine, fasting glucose, and lipid profile levels were assessed using the Beckman Coulter AU5800 clinical chemistry analyzer (Beckman Coulter, Brea, CA, USA). HbA1c levels were measured using the VARIANT II TURBO Hemoglobin Testing System (Bio-Rad Laboratories, Hercules, CA, USA). All analyses were performed according to the instructions from the respective assay manufacturers.

### 2.4. Clinical Data

To assess disease severity and functional outcomes of stroke patients, medical records from when the patient was admitted were collected and reviewed. The following data were collected: symptoms; body mass index (BMI); admission National Institute of Health Stroke Scale (NIHSS) scores, which reflect disease severity; and modified Rankin Scale (mRS) scores at the time of discharge, which reflect the functional outcome.

### 2.5. Statistical Analysis

The Kolmogorov–Smirnov test was conducted to assess the normality of data distribution. Laboratory or clinical data were compared between the study groups using one-way analysis of variance or independent Student's *t*-tests for variables normally distributed and using the Kruskal–Wallis test or Mann–Whitney *U* test for variables not normally distributed. Mann–Whitney *U* tests or Scheffe's multiple comparison tests were conducted for post hoc analysis. Univariable and multivariable logistic regression analyses were conducted to identify biomarkers that could predict ischemic stroke events, while multiple linear regression analysis was used to investigate relationships between laboratory and clinical data. In the logistic regression and multiple linear regression analyses, possible confounding factors such as age; BMI; and serum levels of HbA1c, fasting glucose, and lipids were included, along with serum levels of holoTC, total B12, and homocysteine and clinical scores, to adjust for confounding effects. Statistical analysis was performed using SPSS software, version 19 (SPSS, Chicago, IL), and *P* < 0.05 was considered statistically significant.

## 3. Results

### 3.1. Clinical Characteristics of Stroke Patients

The clinical characteristics of the 130 first-ever ischemic stroke patients are listed in Table 1. The median age of stroke patients was 71 years (interquartile range (IQR), 59–78 years; age range, 44–89 years). The median ages for male and female patients were 67 years (IQR: 58–75 years) and 69 years (IQR, 61–80 years), respectively. The most frequent symptom was left-side hemiparesis/hemiplegia (31.5%), and the most frequently involved brain region was the frontal lobe (23.1%). The median NIHSS scores of the total, male, and female stroke patients were 2 (IQR, 1–5.25; min 0, max 31), 3 (IQR, 1–5.5; min 0, max 31), and 2 (IQR, 0.5–5.5; min 0, max 27), respectively. The median mRS scores of the total, male, and female stroke patients were 4 (IQR, 2–4; min 1, max 5), 4 (IQR, 3–4; min 1, max 5), and 4 (IQR, 2–4; min 1, max 5), respectively. There were no statistically significant differences in the NIHSS and mRS scores between the sexes.

There were no significant differences in age between the sexes in either group or between healthy controls and stroke patients (*P* ≥ 0.05). The median age of healthy controls was 69.8 years (IQR, 62–76 years; range, 46–96 years). The median ages of male and female healthy controls were 67.5 years (IQR, 60–74.5 years) and 70 years (IQR, 64–77 years), respectively.

### 3.2. holoTC, Total B12, and Homocysteine Levels of Study Subjects

Laboratory profiles, including measurements of holoTC, total B12, and homocysteine levels, of stroke patients and healthy controls are listed in Table 2. Absolute serum holoTC levels and holoTC/total B12 ratios were significantly lower in stroke patients than in healthy controls (median: 127.2 vs. 167.4 pmol/L and 33.7% vs. 38.3%, respectively, *P* < 0.01 for both). However, no difference was found in total B12 levels between the two groups (*P* > 0.05). Homocysteine levels were significantly higher in stroke patients than in healthy controls (12.3 vs. 10.2 *μ*mol/L, *P* < 0.01).

The proportions of patients with holoTC and total B12 levels below the cut-off values (<37 pmol/L and <200 pg/mL, respectively) and with hyperhomocysteinemia (>14 *μ*mol/L) were higher in the stroke group than in the control group (holoTC, 6.9% vs. 1.4%; total B12, 10.0 vs. 0.7%; homocysteine, 28.4% vs. 5.1%, respectively; all *P* < 0.05). HbA1c and fasting glucose levels and BMI also differed significantly between the groups (*P* < 0.05).

### 3.3. Logistic Regression Analysis for Predicting Ischemic Stroke

The results of the logistic regression analysis are listed in Table 3. In the univariable logistic regression analysis, holoTC, total B12, holoTC/total B12 ratio, homocysteine, HbA1c, fasting glucose, and BMI showed significant *β* values (*P* < 0.05). Among them, low holoTC/total B12 ratio, high homocysteine and fasting glucose levels, and BMI were statistically significant predictors of ischemic stroke (*P* < 0.05) in the multivariable logistic regression analysis.

### 3.4. Correlations of holoTC and Total B12 Levels with Serum Homocysteine Levels

In Spearman's rank tests, only holoTC levels showed a statistically significant negative correlation with homocysteine levels (*r*_s_, -0.196, *P* = 0.025) in stroke patients. However, in healthy controls, both holoTC and total B12 levels had significant inverse correlations with serum homocysteine levels (*r*_s_, -0.212 and -0.321; *P* = 0.012 and *P* < 0.01, respectively). The holoTC/total B12 ratio did not show a significant correlation with homocysteine levels in patients or healthy controls (both *P* > 0.05). Among stroke patients, more patients had holoTC deficiency with hyperhomocysteinemia (6.2%, 8/130) than total B12 deficiency with hyperhomocysteinemia (3.1%, 4/130).

The multiple linear regression analysis also revealed that holoTC levels had a statistically significant negative correlation with homocysteine levels in patients with stroke (*β* -0.107, *P* < 0.05), but total B12 and holoTC/total B12 levels did not (Table 4). The serum total cholesterol level was positively correlated to homocysteine levels (*P* < 0.05).

### 3.5. Correlations of holoTC, Total B12, and Homocysteine Levels with Functional Scale Scores

Both holoTC and total B12 levels showed significant *r*_s_ values for correlation with NIHSS and mRS scores in Spearman's rank correlation tests after adjusting for HbA1c, fasting glucose, and total cholesterol levels. The *r*_s_ values of the correlation of holoTC and total B12 levels with the NIHSS score were -0.29 and -0.28 (both *P* < 0.01), respectively, and with the mRS scores were -0.31 and -0.29 (both *P* < 0.01), respectively. Homocysteine levels and holoTC/total B12 ratios did not correlate significantly with either of the functional scales (*P* > 0.05).

In contrast, multiple linear regression analyses showed that the holoTC level and holoTC/total B12 ratio were independently and negatively correlated to NIHSS scores (*β*, -0.830 and -0.631; partial *R*, -0.405 and -0.207, respectively, *P* < 0.01 for both), but total B12 and homocysteine levels were not. Fasting glucose, BMI, and patient's age were also associated with NIHSS scores (Table 4). In a subgroup analysis based on the NIHSS score, both holoTC and total B12 levels showed significantly decreased values in the “NIHSS 21–42” subgroup (“severe stroke” patients) compared to any other group (Table 5). The holoTC/total B12 ratio and homocysteine levels were not statistically significantly different among the subgroups. In the mRS subgroup analysis, holoTC levels and total B12 levels were significantly decreased in the “mRS score 5” subgroup compared to any other group (Table 6).

## 4. Discussion

The study findings revealed that the holoTC/total B12 ratio could be a predictive factor for ischemic stroke. holoTC measurements reflected homocysteine levels better than total B12 measurements in stroke patients; thus, holoTC measurements in ischemic stroke patients may be useful for identifying patients who have vitamin B12 deficiency-related hyperhomocysteinemia. In addition, holoTC levels showed significant associations with stroke severity and functional outcomes. To our best knowledge, this was the first case-control study that investigated the diagnostic or prognostic utility of serum holoTC measurements in patients with ischemic stroke.

Vitamin B12 is a cofactor in homocysteine metabolism, and homocysteine can mediate the development of cerebrovascular disease. This can occur through several different mechanisms, including its adverse effects on vascular endothelium and smooth muscle cells, which cause alterations in subclinical arterial structure and function [14–16]. Systematic reviews of observational studies have shown a strong and positive association between serum homocysteine levels and stroke events [5, 17, 18], consistent with our results. Irrespective of increased homocysteine levels, vitamin B12 deficiency has been shown to impair bone metabolism through reduced levels of taurine, an amino acid that is considered to reduce the risks of atherosclerosis and cardiovascular disease, and has shown neuroprotective effects in animal stroke models [9, 19, 20].

Transcobalamin is essential for the transport of vitamin B12 from the intestine into most cells of the body, and patients lacking this protein eventually develop vitamin B12 deficiency [6]. When vitamin B12 is bound to transcobalamin, it is termed holoTC and represents the biologically active fraction of vitamin B12 that can be delivered to all DNA-synthesizing cells. In the absence of a gold standard to detect vitamin B12 deficiency [4, 11], current laboratory tests, which measure total B12, may fail to diagnose the deficiency in the early stage [6, 21, 22]. In 1987, Victor Herbert proposed a model for the staged development of vitamin B12 deficiency, of which holoTC is the first indicator. Based on this model, several studies have confirmed that serum total B12 is a relatively poor marker, with low sensitivity and specificity, for predicting vitamin B12 status, and that instead holoTC may be a useful and sensitive diagnostic indicator of vitamin B12 status [4, 6, 23]. However, Herbert's model for the development of vitamin B12 deficiency is controversial, and an alternative model reflecting the enterohepatic recycling regulation of holoTC was proposed by Golding [11] who argued that holoTC cannot be used to assess vitamin B12 status any more reliably than total B12.

Few studies have investigated holoTC measurements in ischemic stroke patients. One previous study investigated the efficacy of holoTC measurements in ischemic cerebrovascular disease. However, it only focused on changes in holoTC levels before and after treatment with nutritional supplements containing vitamin B12; there was no comparison with healthy controls, the study sample was relatively small (45 patients), and the disease severity or functional outcomes were not assessed [6]. They concluded that the holoTC level was a promising early diagnostic marker of vitamin B12 deficiency, although there was no statistically significant difference in holoTC values reported at the baseline and after vitamin B12 supplementation. The present study showed that serum holoTC levels, holoTC/total B12 ratios, and homocysteine levels differed significantly between ischemic stroke patients and healthy controls. Moreover, the multivariable logistic regression analysis revealed that holoTC/total B12 ratios and homocysteine levels may be used as predictive factors for the development of ischemic stroke. Taken together, our findings implied that holoTC and total B12 levels could be valuable biomarkers for predicting ischemic stroke risk, along with homocysteine and total B12 levels.

Despite ongoing controversy, several findings in the current study may support the use of holoTC over total B12 measurements in patients with first-ever ischemic stroke. First, holoTC levels were lower in patients than in healthy controls, in both male and female subgroups, and homocysteine levels were also significantly higher in stroke patients of both sexes. However, when total B12 levels were considered, these differences were only apparent in men. Second, in stroke patients, homocysteine levels were inversely associated with holoTC levels but not with total B12 levels based on both of Spearman's rank test and multiple linear regression analysis. Finally, among patients with hyperhomocysteinemia, the holoTC level was decreased in around 21.6% of patients, while the total B12 level was decreased in only 10.8% of patients. Taken together, holoTC measurements are likely to better reflect homocysteine levels than total B12 measurements. As high levels of homocysteine in ischemic stroke patients can lead to recurrent ischemic stroke events [24], holoTC measurements in ischemic stroke patients may be useful for identifying patients who require vitamin B12 supplementation.

The holoTC/total B12 ratio was an independent predictive factor for ischemic stroke, but holoTC and total B12 levels alone were not. Garrod et al. reported that the holoTC/total B12 ratio was associated with cognitive function in old patients with depressive symptoms, and they concluded that the holoTC/total B12 ratio may better reflect the adequacy of B12 for nervous system function than holoTC or total B12 level alone [25, 26]. However, ischemic stroke is a vascular event, and we did not find any evidence about the relationship between holoTC/total B12 ratios and homocysteine levels. It was plausible that the holoTC/total B12 ratio reflected B12 deficiency in the endovascular system more accurately than the total B12 or holoTC level alone, but the exact mechanisms are unclear. The holoTC/total B12 ratio was also inversely correlated to disease severity in the multiple linear regression analyses. Because serum folate and vitamin B6 levels could also affect homocysteine levels [27] and vitamin B12 deficiency has the potency to reduce taurine levels as described above [9, 19, 20], further investigation with these biomarkers needs to be performed to explore the utility of holoTC/total B12 ratio measurement and its related mechanisms in stroke patients.

Both holoTC (in multiple linear regression analysis and Spearman's rank test) and total B12 (in Spearman's rank test only) levels, but not serum homocysteine levels, showed significant correlations with the NIHSS score. Additionally, quantitative comparisons between subgroups stratified by the NIHSS score showed that the subgroups with NIHSS scores of 21–42 had significantly lower holoTC and total B12 levels than other subgroups, whereas no differences were found with respect to homocysteine levels. Although results of several previous studies are concordant with this result [28, 29], most studies concluded that there was a significant correlation between disease severity and homocysteine levels [2, 30, 31]. The reasons for differences between our own findings and those of previous studies and for the correlation of holoTC and vitamin B12 levels, but not homocysteine levels, with disease severity are unclear. One possible cause for these discrepancies is the relatively small number of patients with NIHSS scores ≥ 16 in this study. Almost all patients (92%) had NIHSS scores ≤ 15, which could have affected the disease severity results. Vitamin B12 levels may have direct effects on the peripheral or central nervous systems, including cellular energetic functions, antioxidative and neuroprotective actions, and both myelin and neurotransmitter synthesis [32]. These could have affected disease severity irrespective of homocysteine levels, even if the effects were small. The dynamics of homocysteine levels during the disease course could be another cause. Homocysteine levels can fluctuate or increase 24 hours after stroke onset [33]. Because homocysteine is an acute phase reactant, the possibility of the acute phase response being responsible for the elevation in serum homocysteine levels in acute stroke patients cannot be ruled out. Thus, it is very important to be careful when interpreting results related to homocysteine levels.

Similar to the NIHSS score, the mRS score at the time of discharge was correlated with holoTC and total B12 levels, but not with homocysteine levels. These findings were concordant with results of most previous studies [2, 34, 35]. Markisic et al. revealed that worse functional outcomes, assessed according to the mRS after a 6-month follow-up, only correlated with total B12 measurements, and hyperhomocysteinemia did not correlate with mRS scores at any follow-up time point [2]. Mizrahi et al. also reported no correlations between homocysteine levels and functional outcomes when the functional independence measure tool was used [35]. Contrary to most studies, one study reported that the combination of homocysteine and high-sensitivity C-reactive protein levels was associated with short-term outcomes of ischemic stroke [36].

This study had several limitations. First, because of the lack of related medical records, we did not assess physical activity, alcohol overuse, unhealthy diet, psychosocial stress, or depression, which are factors known to contribute to ischemic stroke risk [2]. Second, serum levels of vitamin B9 (folic acid) and vitamin B6, which can also affect homocysteine levels, were not measured. In addition, serum levels of cytokines such as interleukins and tumor necrosis factors, which can be confounding factors of homocysteine levels [37], were not measured. They were also known to be related to disease severity [38]. We also did not assess the levels of methylmalonic acid, which is another biomarker affected by vitamin B12 deficiency. Third, we did not investigate point mutations in the coding region of the *MTHFR* gene, which is associated with mild and moderate hyperhomocysteinemia [39]. Fourth, the number of patients with severe ischemic stroke was relatively small. Finally, the onset time of stroke symptoms mainly depended on patients' or their families' recall; thus, it could not be validated.

## 5. Conclusion

Despite the limitations, our findings suggested that measurements of serum holoTC along with total B12 and homocysteine may provide valuable information for predicting ischemic stroke onset and its severity and short-term outcomes of ischemic stroke patients. Further research is needed for improved validation.

## Figures and Tables

**Table 1:** Characteristics of the first-ever stroke patients in this study.

Characteristics	Patients with ischemic stroke (*N* = 130)
Age, median years [Q1–Q3]	71 [59–78]
Sex, % male (*N*)	62.3 (81)
Clinical presentations, % (*N*)	
Hemiparesis/hemiplegia/weakness	
Left	31.5 (41)
Right	16.2 (21)
Bilateral	1.5 (2)
Dysarthria	27.7 (36)
Dizziness	16.2 (21)
Mental change	10.0 (13)
Amnesia	1.5 (2)
Involved brain region, % (*N*)	
Frontal lobe	23.1 (30)
Temporal lobe	22.3 (29)
Parietal lobe	16.2 (21)
Occipital lobe	12.3 (16)
Basal ganglia	14.6 (19)
Corona radiata	13.8 (18)
Pons	13.1 (17)
Cerebellum	17.7 (23)
Brain atrophy, % (*N*)	6.9 (9)
NIHSS, median [Q1–Q3]	2 [1–5.25]
mRS, median [Q1–Q3]	4 [2–4]

Abbreviations: NIHSS: National Institute of Health Stroke Scale; mRS: modified Rankin Scale.

**Table 2:** Comparison of holotranscobalamin (holoTC), total vitamin B12, homocysteine, glucose markers, lipids levels, and body mass index between stroke patients and healthy controls according to sex.

	Total			Male			Female		
	Stroke (*N* = 130)	Control (*N* = 138)	*P* ^∗^	Stroke (*N* = 81)	Control (*N* = 88)	*P* ^∗^	Stroke (*N* = 49)	Control (*N* = 50)	*P* ^∗^
holoTC (pmol/L)	127.2^†^ [89–193.5]	167.4 [120.6–225]	<0.01	125.6 [87.25–177.75]	158.1 [113.2–209.8]	<0.01	137.4 [25.8–83.2]	185.8 [131.2–232.5]	0.04
Proportion of holoTC < 37 pmol/L, % (*N*)	6.9 (9)	1.4 (1)	<0.01	7.4 (6)	1.1 (1)	0.04	6.1 (3)	0.0 (0)	<0.01
Total vitamin B12 (pg/mL)	521.5 [384–781]	619.5 [451–764]	0.07	487 [377–666]	667 [452.5–812.5]	<0.01	535 [385–787.5]	577.5 [470–684]	0.16
Proportion of total vitamin B12 < 200 pg/mL, % (*N*)	10.0 (13)	0.7 (1)	<0.01	7.4 (6)	1.1 (1)	0.04	14.3 (7)	0.0 (0)	<0.01
holoTC/total vitamin B12 ratio (%)^‡^	33.7 [26.6–40.3]	38.3 [31.3–45.7]	<0.01	33.8 [26.4–42.9]	39.2 [32.8–45.9]	0.04	32.9 [27.6–38.3]	35.9 [29.7–45.8]	0.03
Homocysteine (*μ*mol/L)	12.3 [10.2–14.4]	10.2 [8.47–11.8]	<0.01	12.61 [10.4–15.3]	10.9 [8.2–11.7]	<0.01	11.7 [9.7–13.4]	10.3 [8.6–11.9]	0.03
Proportion of homocysteine > 14 *μ*mol/L, % (*N*)	28.4 (37)	5.1 (7)	<0.01	35.8 (29)	6.8 (6)	<0.01	16.3 (8)	2.0 (1)	0.01
Hemoglobin A1c (%)	5.75 [5.4–6.7]	5.6 [5.4–6.0]	0.01	5.9 [5.5–7.1]	5.6 [5.3–6.1]	<0.01	5.6 [5.2–6.2]	5.7 [5.5–5.8]	0.25
Proportion of hemoglobin A1c > 6.5%, % (*N*)	30.0 (39)	7.2 (10)	<0.01	35.8 (29)	11.4 (10)	<0.01	20.4 (10)	0.0 (0)	<0.01
Fasting glucose (mg/dL)	109 [94–145]	104.5 [95–116]	0.01	115 [97.5–154.5]	104.5 [97–115.5]	0.01	101 [89.5–131.5]	104 [91–119]	0.68
Proportion of fasting glucose > 126 mg/dL, % (*N*)	36.2 (47)	14.5 (20)	<0.01	42.0 (34)	14.8 (13)	<0.01	26.5 (13)	14.0 (7)	0.12
Triglyceride (mg/dL)	101 [69–132]	101 [68–132]	0.69	116 [79.5–171.5]	110.5 [73.5–140]	0.52	88 [57.5–118]	85.5 [68–125]	0.85
LDL cholesterol (mg/dL)	105.8 ± 66.6^†^	99.0 ± 62.0	0.13	111.2 ± 60.7	101.2 ± 64.0	0.08	90.8 ± 68.0	93.5 ± 56.4	0.65
Total cholesterol (mg/dL)	176.8 ± 92.8	164.8 ± 89.8	0.05	182.5 ± 86.9	167.5 ± 93.2	0.04	166.3 ± 91.2	161.4 ± 80.4	0.56
BMI (kg/m^2^)	24.9 ± 6.6	23.6 ± 5.8	<0.01	25.5 ± 5.7	23.6 ± 5.0	<0.01	24.2 ± 9.0	2.5 ± 7.2	0.30
Proportion of BMI > 25 kg/m^2^, % (*N*)	31.5 (41)	26.8 (37)	0.39	35.8 (29)	25.0 (22)	0.13	24.5 (12)	30.0 (15)	0.53

Abbreviations: holoTC: holotranscobalamin; LDL: low-density lipoprotein; BMI: body mass index. ^∗^Calculated from Mann–Whitney *U* test or independent Student's *t*-test according to data distribution. ^†^Data are displayed as “median [Q1–Q3]” or “mean ± 2 standard deviations” according to their distribution. ^‡^Calculated after unifying the units for total vitamin B12 and holotranscobalamin.

**Table 3:** Logistic regression analysis results for predicting ischemic stroke.

Variables	Univariable			Multivariable		
	*β* coefficient (SE)	OR (95% CI)	*P*	*β* coefficient (SE)	OR (95% CI)	*P*
holoTC (pmol/L)	-0.004 (0.002)	0.996 (0.993–0.999)	<0.01	—	—	>0.05
Total vitamin B12 (pg/mL)	-0.001 (0.001)	0.999 (0.998–1.000)	0.01	—	—	>0.05
holoTC/total vitamin B12 (%)^∗^	-0.410 (0.011)	0.960 (0.938–0.981)	<0.01	-0.450 (0.013)	0.956 (0.932–0.981)	<0.01
Homocysteine (*μ*mol/L)	0.270 (0.049)	1.310 (1.190–1.442)	<0.01	0.251 (0.051)	1.285 (1.163–1.421)	<0.01
Hemoglobin A1c (%)	0.503 (0.133)	1.653 (1.274–2.144)	<0.01	—	—	>0.05
Fasting glucose (mg/dL)	0.014 (0.004)	1.014 (1.006–1.021)	<0.01	0.015 (0.004)	1.015 (1.007–1.024)	<0.01
Triglyceride (mg/dL)	—	—	>0.05	—	—	—
LDL cholesterol (mg/dL)	—	—	>0.05	—	—	—
Total cholesterol (mg/dL)	—	—	>0.05	—	—	—
BMI (kg/m^2^)	0.152 (0.042)	1.164 (1.073–1.263)	<0.01	0.118 (0.046)	1.125 (1.029–1.231)	0.01

Abbreviations: SE: standard error; CI: confidence interval; holoTC: holotranscobalamin; LDL: low-density lipoprotein; BMI: body mass index. ^∗^Calculated after unifying the units for total vitamin B12 and holotranscobalamin.

**Table 4:** Association of metabolic variables with serum homocysteine levels and NIHSS by multiple linear regression analyses in 130 patients with first-ever ischemic stroke.

Variables	Homocysteine (*μ*mol/L)			NIHSS		
	*β* (SE)	Partial *R*	*P*	*β* (SE)	Partial *R*	*P*
holoTC (pmol/L)^∗^	-0.107 (0.054)	-0.165	0.048	-0.830 (0.156)	-0.405	<0.01
Total vitamin B12 (pg/mL)^∗^	—	—	—	—	—	—
holoTC/total vitamin B12 (%)^∗^^,†^	—	—	—	-0.631 (0.233)	-0.207	<0.01
Homocysteine (*μ*mol/L)^∗^				—	—	—
Hemoglobin A1c (%)^∗^	—	—	—	—	—	—
Fasting glucose (mg/dL)^∗^	—	—	—	0.532 (0.223)	0.182	0.019
Triglyceride (mg/dL)^∗^	—	—	—	—	—	—
LDL cholesterol (mg/dL)	—	—	—	—	—	—
Total cholesterol (mg/dL)	0.004 (0.001)	0.179	0.040	—	—	—
BMI (kg/m^2^)	—	—	—	0.022 (0.011)	0.158	0.041
Age (years)^∗^	—	—	—	0.006 (0.003)	0.146	0.049

Abbreviations: SE: standard error; holoTC: holotranscobalamin; LDL: low-density lipoprotein; BMI: body mass index. ^∗^Log-transformed values were used for analysis. ^†^Calculated after unifying the units for total vitamin B12 and holotranscobalamin.

**Table 5:** Holotranscobalamin (holoTC), total vitamin B12, homocysteine, glucose markers, lipids levels, and body mass index according to the National Institute of Health Stroke Scale (NIHSS).

	NIHSS					*P* ^∗^	Kruskal–Wallis grouping^†^
	0 (*N* = 26)	1–4 (*N* = 65)	5–15 (*N* = 29)	16–20 (*N* = 4)	21–42 (*N* = 6)		
holoTC (pmol/L)	143.7^‡^ [73.0–184.8]	127.0 [102.8–196.5]	118 [77.2–178.8]	141 [117–156]	58.1 [32.0–62.4]	0.04	a/b/b/a/c
Total vitamin B12 (pg/mL)	496.5 [280.0–679.5]	558 [440–819.3]	480 [299.0–756.0]	393 [277–566]	194 [162–290]	<0.01	a/b/a/c/d
holoTC/total vitamin B12 (%)^§^	34.0 [30.8–41.9]	30.6 [24.7–38.4]	34.5 [30.4–42.4]	43.7 [26.1–62.2]	29.5 [27.9–48.0]	0.143	
Homocysteine (*μ*mol/L)	11.7 [9.7–13.3]	12.6 [10.8–14.7]	12.8 [9.7–14.4]	13 [11.0–15.8]	10.9 [10.4–11.2]	0.26	—
Hemoglobin A1c (%)	5.5 [2.3–5.7]	5.9 [5.5–6.9]	6.3 [5.6–7.4]	6 [5.5–6.0]	5.5 [5.0–6.2]	0.02	a/b/b/b/a
Fasting glucose (mg/dL)	100.0 [92.3–110.0]	109.0 [94–135.8]	112.0 [99.0–159.0]	130 [112.0–147.8]	189.5 [156.5–209.0]	0.04	a/a/a/a/b
Triglyceride (mg/dL)	81 [54.0–107.5]	116 [79.0–171.0]	101.0 [52.0–129.0]	102 [53.5–160.5]	108.5 [69.8–145.8]	0.11	—
LDL cholesterol (mg/dL)	101.7 ± 31.6^‡^	103.5 ± 36.02	106.5 ± 31.7	85.8 ± 34.5	108.8 ± 21.5	0.71	—
Total cholesterol (mg/dL)	174.0 ± 85.9	181.6 ± 83.6	174.6 ± 88.9	164.3 ± 69.6	169.2 ± 58.9	0.64	—
BMI (kg/m^2^)	25.1 ± 6.7	25.3 ± 6.9	24.3 ± 5.0	22.3 ± 7.2	27.0 ± 8.7	0.57	—

Abbreviations: LDL: low-density lipoprotein; BMI: body mass index. ^∗^Calculated from the one-way analysis of variance or the Kruskal–Wallis test, according to data distribution. ^†^The same letters indicate the nonsignificant difference between groups based on the Mann–Whitney *U* test. ^‡^Data are displayed as “median [Q1–Q3]” or “mean ± 2 standard deviations” according to their distribution. ^§‡^Calculated after unifying the units for total vitamin B12 and holotranscobalamin.

**Table 6:** Holotranscobalamin (holoTC), total vitamin B12, homocysteine, glucose markers, lipids levels, and body mass index levels according to the modified Rankin Scale (mRS).

	mRS					*P* ^∗^	Kruskal–Wallis grouping^†^
	1 (*N* = 13)	2 (*N* = 21)	3 (*N* = 17)	4 (*N* = 56)	5 (*N* = 23)		
holoTC (pmol/L)	139.6^‡^ [102.8–200.0]	137.4 [108.2–207.2]	108.0 [84.4–190.1]	109.1 [75.7–176.2]	72.7 [58.1–128.1]	<0.01	a/a/a/a/b
Total vitamin B12 (pg/mL)	558.0 [376.0–814.0]	556.0 [475.3–787.8]	430.0 [361.0–770.5]	486.0 [288.0–631.0]	302.0 [86.5–502.5]	<0.01	a/a/a/a/b
holoTC/total vitamin B12 (%)^§^	35.6 [29.5—42.6]	31.8 [27.8–38.3]	33.6 [28.7–37.6]	33.9 [25.2–43.3]	31.0 [26.0–40.8]	0.84	**—**
Homocysteine (*μ*mol/L)	11.2 [10.0–17.4]	12.8 [9.8–13.9]	13.3 [10.9–15.1]	12.3 [10.2–14.7]	11.9 [9.8–14.6]	0.94	—
Hemoglobin A1c (%)	5.7 [5.1–7.6]	6.1 [5.4–7.0]	6.1 [5.6–8.0]	5.8 [5.3–6.7]	5.7 [5.4–6.4]	0.44	—
Fasting glucose (mg/dL)	143.0 [104.0–207.0]	107.0 [92.0–150.5]	123.0 [104.0–161.0]	107.0 [91.0–137.8]	105.0 [96.0–130.0]	0.14	—
Triglyceride (mg/dL)	60.0 [52.0–142.0]	88.0 [50.5–135.5]	104.0 [76.0–143.0]	99.5 [70.0–183.8]	112.0 [88.0–171.0]	0.40	—
LDL cholesterol (mg/dL)	107.4 ± 76.4^‡^	94.3 ± 67.4	108.5 ± 46.3	105.8 ± 74.8	100.1 ± 52.1	0.67	—
Total cholesterol (mg/dL)	178.5 ± 95.4	159.1 ± 102.1	181.5 ± 67.9	181.2 ± 101.7	167.6 ± 72.6	0.42	—
BMI (kg/m^2^)	24.7 ± 3.2	24.3 ± 8.2	23.9 ± 4.6	25.7 ± 6.9	25.0 ± 5.5	0.17	—

Abbreviations: LDL: low-density lipoprotein; BMI: body mass index. ^∗^Calculated from the one-way analysis of variance or the Kruskal–Wallis test according to data distribution. ^†^The same letters indicate the nonsignificant difference between groups based on the Mann–Whitney *U* test. ^‡^Data are displayed as “median [Q1, Q3]” or “mean ± 2 standard deviations” according to their distribution. ^§‡^Calculated after unifying the units for total vitamin B12 and holotranscobalamin.

## Data Availability

The data used to support the findings of this study are available from the corresponding author upon request.
